# The mitochondrial genome of a parasitic wasp, *Chouioia cunea* Yang (Hymenoptera: Chalcidoidea: Eulophidae) and phylogenetic analysis

**DOI:** 10.1080/23802359.2021.1886008

**Published:** 2021-03-15

**Authors:** Xue Tang, Baoqian Lyu, Hui Lu, Jihong Tang, Rui Meng, Bo Cai

**Affiliations:** a Environment and Plant Protection Institute, China Academy of Tropical Agriculture Sciences, Haikou, China; b Key Laboratory of Integrated Pest Management on Tropical Crops, Ministry of Agriculture and Rural Affairs, Haikou, China; c Provincial Key Laboratory for Agricultural Pest Management of Mountainous Regions, Institute of Entomology, Guizhou University, Guiyang, China; d Post-Entry Quarantine Station for Tropical Plant, Haikou Customs, Haikou, China; e Hainan Province Engineering Research Center for Quarantine, Prevention and Control of Exotic Pests, Haikou, China

**Keywords:** Chalcidoidea, gene rearrangement, mitochondrial genome, *Chouioia cunea* Yang, phylogenetic relationship

## Abstract

*Chouioia cunea* Yang 1989 is a parasitic wasp and natural enemy of several lepidopteran pests during their pupal stage. In this study, we sequenced and analyzed the mitochondrial genome of *C. cunea*, and obtained a complete DNA molecule that is 14,930 bp in size with 13 protein-coding genes (PCGs), two ribosomal RNA genes (rRNAs), and 22 transfer RNA genes (tRNAs) (GenBank accession number MW192646). All the 13 PCGs started with typical ATN (ATA, ATG, and ATT) and terminated with the stop codon TAA or TAG. Phylogenetic analysis showed that *C. cunea* formed the sister group with *Tamarixia radiata*, which belongs to the same family.

*Chouioia cunea* Yang (Eulophidae: Chalcidoidea) was first collected from pupal of the fall webworm (*Hyphantria cunea* Drury), which can also be used for biological control of a variety of Lepidopteran pests such as *c* Fabricius, *Clania variegata* Snellen, *Stilpnotia candida* Staudinger, *S. salicis* (L.), *Micromilalopha troglodyta* (Graeser), and *Ivela ochropoda* Fabricius (Zhao et al. 2016; Xin et al. 2017). Insect mitochondrial genome has many characteristics which can make it play an important role in molecular evolution, phylogenetics, and population genetics. However, only *T. radiata* has been sequenced in Eulophidae (Du et al. 2019).

In this study, individuals of *C. cunea* were collected from a field in Hainan province and reared in the Environment and Plant Protection Institute, China Academy of Tropical Agriculture Sciences, Hainan, China (110°20′9″N, 19°59′21″E). The samples were preserved in 95% ethanol at −20 °C in herbarium of Post-Entry Quarantine Station for Tropical Plant, Haikou Customs District, PR China with an accession number IN07040201-0001-0020. Single sample were used for genomic DNA extraction. The mitogenome sequence of *C. cunea* was generated using Illumina HiSeq X TEN Sequencing System and assembled by MitoZ software without parameters (Meng et al. 2019).

The mitochondrial genome of *C. cunea* is 14,930 bp long, including 13 protein-coding genes (PCGs), 22 transfer RNA genes (tRNAs), two ribosomal RNA genes (rRNAs), and one partial non-coding AT-rich region with a length of 220 bp. All the genes were distributed on two coding chains, of which 27 genes were encoded on majority strand (J-chains) and the rest were transcribed on minority chains (N-chains). The overall base composition of the mitogenome sequence is 44.8% A, 40.3% T, 8.2% C, 6.7% G, with a high AT bias of 85.1%. Compared with ancestral insect mitochondrial genome, the mitogenome of *C. cunea* exhibits a dramatic mitochondrial gene rearrangement, of which is consistent with previous studies (Wu et al. 2020). A total of 27 genes have been rearranged in the *C. cunea*, including seven PCGs (*trnC*, *trnK*, *trnD*, *ATP8*, *ATP6*, *NADH3*, *trnR*), 20 tRNAs. The gene block (NADH3-trnG-COX3-ATP6-ATP8-trnD-trnQ-trnK-COX2-trnL2-COX1) was inverted in the mitochondrial genome of *C. cunea*.

All the 13 PCGs of *C. cunea* started with the conventional ATN codons, including three ATAs (*ND1*, *ND3*, and *ND4L*), five ATTs (*ND2*, *ND5*, *ND6*, *COX2*, and *ATP8*), five ATGs (*COX1*, *ND4*, *CYTB*, *COX3*, and *ATP6*). Twelve PCGs terminate with the stop codon TAA, whereas *ND1* end with TAG. The length of 22 tRNA genes is between 58 and 69 bp, and all them have the typical clover-leaf structure, except for trnS1 and trnR which lack dihydrouridine (DHU) arm. It is a common phenomenon that trnS1 lacks DHU arm in the mitochondrial genome of many insects (Yuan et al. 2015; Xiong et al. 2019). Two rRNA genes (s-rRNA and l-rRNA) located at trnV/trnA and trnA/trnL1 regions, with the lengths of 754 and 1331 bp, respectively.

To validate the phylogenetic status of *C. cunea*, we selected the mitochondrial DNA sequences of 18 closely related taxa of Chalcidoidea in NCBI, and extracted the sequences by Phylosuite software (Zhang et al. 2020). *Pelecinus polyturator* and *Ibalia leucospoides* were used as outgroups. The analyses were performed with Bayesian inference and maximum likelihood in Phylosuite (Nguyen et al. 2015; Zhang et al. 2020). The result showed that *C. cunea* was clustered with *T. radiata* (Figure 1), which belongs to the same family. To sum up, the results of this study can provide essential and important DNA molecular data for further phylogenetic and evolutionary analysis of Chalcidoidea.

**Figure 1.**
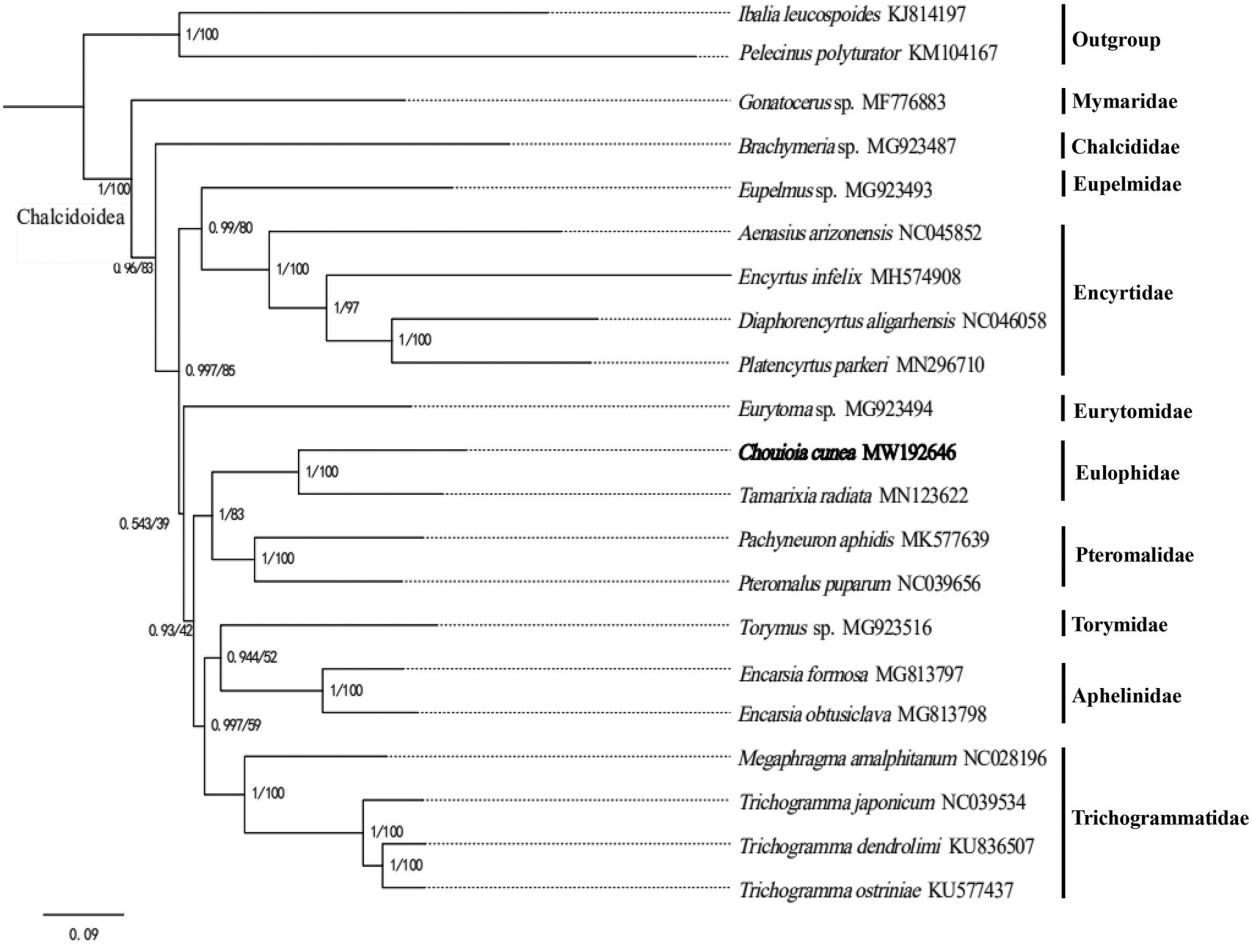
Phylogenetic relationships of 13 mitochondrial protein-coding genes sequences within Chalcidoidea were performed using Bayesian/ML methods.

## Data Availability

The genome sequence data that support the findings of this study are openly available in GenBank of NCBI at https://www.ncbi.nlm.nih.gov/ under the accession no. MW192646. The associated BioProject, SRA, and Bio-Sample numbers are PRJNA691215, SRP301245, and SRS8004285, respectively.
